# Further characterization and engineering of an 11-amino acid motif for enhancing recombinant soluble protein expression

**DOI:** 10.1186/s12934-025-02738-5

**Published:** 2025-05-24

**Authors:** Jiawu Bi, Elaine Tiong, Ying Sin Koo, Weibiao Zhou, Fong Tian Wong

**Affiliations:** 1 Institute of Molecular and Cell Biology (IMCB), Agency for Science, Technology and Research (A*STAR), 61 Biopolis Drive, Proteos #07-06, Singapore, 138673 Republic of Singapore; 2 Department of Food Science and Technology, National University of Singapore (NUS), Faculty of Science, 2 Science Drive 2, Singapore, 117542 Republic of Singapore; 3 Institute of Sustainability for Chemicals, Energy and Environment (ISCE2), Agency for Science, Technology and Research (A*STAR), 8 Biomedical Grove, Neuros, #07-01, Singapore, 138665 Republic of Singapore

**Keywords:** Protein solubility tag, Growth factor, Enzyme, Recombinant protein expression, *Escherichia coli*

## Abstract

**Background:**

*Escherichia coli* (*E. coli*) is a popular system for recombinant protein production, owing to its low cost and availability of genetic tools. However, the expression of soluble recombinant proteins remains an issue. As such, various solubility-enhancing and yield-improving methods such as the addition of fusion tags have been developed. This study focuses on a small solubility tag (NT11), derived from the N-terminal domain of a duplicated carbonic anhydrase from *Dunaliella* species. The small size of NT11 (< 10 kDa) lowers the chance of protein folding interference and post-translation removal requirement, which ultimately minimizes cost of production.

**Results:**

A comprehensive analysis was performed to improve the characteristics of the 11-amino acid tag. By investigating the alanine-scan library of NT11, we achieved at least a two-fold increase in protein yield for three different proteins and identified key residues for further development. We also demonstrated that the NT11 tag is not limited to the N-terminal position and can function at either the N- or C-terminal of the protein, providing flexibility in designing constructs. With these new insights, we have successfully doubled the recombinant soluble protein yields of valuable growth factors, such as fibroblast growth factor 2 (FGF2) and human epidermal growth factor (hEGF) in *E. coli.*

**Conclusion:**

The further characterisation and development of the NT11 tag have provided valuable insights into the optimisation process for such small tags and expanded our understanding of its potential applications. The ability of the NT11 tag to be positioned at either the N- or C- termini within the protein construct without compromising its effectiveness to enhance soluble recombinant protein yields, makes it a valuable tool across a diverse range of proteins. Collectively, these findings demonstrate a promising approach to simplify and enhance the efficiency of soluble recombinant protein production.

**Supplementary Information:**

The online version contains supplementary material available at 10.1186/s12934-025-02738-5.

## Background

*Escherichia coli* is commonly used for producing recombinant proteins in both academic and industrial settings. It is well-researched, grows quickly, and can be easily cultured in large quantities [1]. Protocols for using *E. coli* are cost-effective and offer a wide range of strains, reagents, promoters, and tools for producing functional proteins. While this recombinant protein system generally works well for microbial proteins, the protein translational environment within the microbial cells is often not suitable for various mammalian derived proteins, ranging from enzymes to growth factors. Research indicates that only 25% of human proteins can be expressed in *E. coli* in an active soluble form [2, 3], with most proteins encountering difficulties during expression—resulting in insoluble aggregates or non-functional peptides [3, 4]. While the simplicity of the bacterial system makes it easy to use, it presents challenges for expressing difficult proteins. Soluble recombinant protein expression may fail due to improper disulfide bond formation, lack of necessary chaperones, or the host's inability to perform required post-translational modifications [5, 6].

To improve protein yields, gene fusion and solubility tags technology are often used. Traditional fusion solubility tags such as N utilization substance protein A (NusA), Glutathione S-transferase (GST), Maltose binding protein (MBP), and small ubiquitin-related modifier (SUMO) are employed to facilitate the folding and solubilization of recombinant proteins [7–11]. While these tags can improve the solubility and yield of fusion proteins [8–12], their large sizes may disrupt the folding and structural integrity of the fusion partners. This results in the production of soluble but non-functional or less active proteins [5, 6, 13], illustrating the limitations of these traditional solubility tags [7, 12]. Therefore, this highlights the need to discover and develop new tags to complement existing ones, especially with the increasing use of microbial systems for protein expression.

More recently, shorter and smaller motifs have been discovered to enhance solubility. One example is the Fh8 fusion tag (8 kDa) and its derivative, the H tag (the first 11 amino acids of Fh8), which can serve as a purification tag, but also show high solubility, thermostability and increased protein expression in *E. coli* when attached to a protein partner. Compared to larger tags, smaller tags could potentially have larger scopes of application due to its inherent lower metabolic stress imposed on the host and a reduced likelihood of interfering with the activity of the expressed protein [11, 14]. Protein tags are now developed with more functionalities and flexibility in mind, offering novel solutions and expanding the design space for producing heterologous proteins in microbial systems.

In this study, we focus on an 11-amino acid protein motif named NT11 [15], which enhances protein solubility and yield in *E. coli* expression systems. The NT11-tag was originally derived from the first 11 amino acid residues within the N-terminal N-half domain of a duplicated carbonic anhydrase (dCA) from *Dunaliella* algae species. The carbonic anhydrase enzyme consists of two main domains: the N-terminal domain and the C-terminal domain. When expressed individually, the N-terminal domain is highly soluble but lacks enzymatic activity, whereas the C-terminal domain is highly insoluble yet retains its enzymatic function. Further experiments then identified the minimal peptide length of 11 residues from the N-terminal domain (NT11), which was then adopted as a short acidic peptide tag capable of improving protein solubility and expression levels without interfering with protein structure or activity [15]. Previously, we employed this tag to successfully solubilize and recover active proteins from a previously insoluble halogenase, as well as mined PET-degrading enzymes [16, 17].

Here, we conducted a detailed characterization of the 11-amino acid motif to gain better insights and improvement of the small but powerful tag. We first focused on examining the alanine-scan library of NT11 to enhance its activity and determine key residues for further engineering. Although the final optimized tag design varies depending on the protein of interest, screening with the alanine mutant library consistently resulted in at least a two-fold improvement in protein yield for three different proteins. Further investigation of NT11 also revealed new characteristics, where we demonstrate for the first time that its impact on enhancing protein production is not limited to a traditional N-terminal position; NT11 can function at either the N- or C-terminal of the protein of interest. This characteristic offers potential flexibility in designing the final protein expression constructs, allowing us to consider both solubility and purification aspects which ultimately expands tag functionality. Overall, further characterization and engineering of the NT11 tag have provided insights into the optimisation process of such small tags and expanded on the understanding of how the tag can be used. With these new insights, we have managed to enhance protein yields of high value growth factors, including fibroblast growth factor 2 (FGF2) and human epidermal growth factor (hEGF), in *E. coli*.

## Methods

### Construction of protein expression vectors and strains

Genes were codon-optimised for *E. coli* and synthesized from Twist Bioscience for assembly into pET28a (+) plasmids using *E. coli* OmniMax (Thermo Fisher Scientific, USA). *E. coli* XJb (DE3) (Zymo Research, USA) was used for protein expression. The amino acid sequences for the protein constructs are documented in Table S1.

### Mutagenesis

Mutagenesis of NT11 tag was generated using the QuikChange™ Site-Directed Mutagenesis System developed by Strategene (La Jolla, CA, USA), with short complementary primers (IDT, Singapore). The primers used for QuikChange™ are documented in Table S2.

### Protein expression

An initial rapid high-throughput screen of the NT11 alanine scan library was conducted with eGFP, LCC-ICCG and FAST PETase. Single colonies (*E. coli* XJb (DE3)) were cultured in 1 mL of overnight autoinduction media (Overnight Express™ Autoinduction Systems, Merck, USA) supplemented with 50 µg/mL kanamycin, 1.5 M l-arabinose, and 0.5 M magnesium chloride. After 20 h of culturing at 28 °C, the samples were pelleted at 21,000 g.

Experiments with LCC-ICCG and brazzein used a two-step process: (1) day 1 single colonies were inoculated into 1 mL Luria Bertani (LB) broth, containing 50 μg/mL kanamycin (LB + kanamycin), overnight at 37 °C, then (2) equal densities were seeded into 50 mL LB + kanamycin, grown to 0.4–0.6 OD600, induced with 100 μM isopropyl-β-d-1-thiogalactopyranoside (IPTG), and cultured at 30 °C for 20 h before pelleting at 4000 g.

Growth factor expression used a day 1 LB with kanamycin starter culture, followed by day 2 inoculation into 1 mL overnight autoinduction media (Overnight Express™ Autoinduction Systems, Merck, USA) with 50 µg/mL kanamycin, 1.5 M L-arabinose, and 0.5 M magnesium chloride, cultured in a BioLector XT Microbioreactor (Beckman Coulter, USA) at 30 °C, 1400 rpm, 25 mL/min airflow for 20 h before pelleting at 21,000 g.

### Protein purification

For all 1 mL protein expressions, the harvested cell pellets underwent a single freeze–thaw cycle in lysis buffer (50 mM sodium phosphate, 300 mM sodium chloride, 10 mM imidazole, 0.03% Triton X-100). The His-tagged proteins were purified via immobilized metal affinity chromatography (IMAC) using Ni–NTA resin (HisPur™ Ni–NTA resin 88222, Thermo Fisher Scientific, USA), then eluted in 50 mM sodium phosphate, 300 mM sodium chloride, 500 mM imidazole.

For 50 mL protein expressions, the harvested pellets were resuspended in 10 mL lysis buffer, sonicated at 20% amplitude (10 s/30 s on/off for 5 min 40 s, Qsonica, USA), and clarified by 13,000 g centrifugation. The soluble His-tagged proteins were then captured on Ni–NTA resin (HisPur™ Ni–NTA resin, 88,222, Thermo Fisher Scientific, USA) and eluted in the same elution buffer as the 1 mL scale. Gravity flow columns (Pierce, Thermo Scientific, USA) were used for affinity chromatography.

### Quantitation of protein expression

Purified proteins were eluted in volumes suited to their scale: 100 μL for 1 mL high-throughput, 2 mL for 50 mL shake flasks, 1 mL for 1 mL BioLector XT microbioreactor (Beckman Coulter, Germany). 10 μL of each elute was mixed with Laemmli buffer (2 × Laemmli Sample Buffer, #1610737EDU, Bio-Rad, USA), boiled at 95 °C for 5 min, and run on SDS-PAGE gels (Thermo Fisher Scientific, USA). The gels were stained for ≥ 30 min with InstantBlue^®^ Coomassie Protein Stain (Abcam, UK). Protein yields were quantified by densitometric analysis using ImageJ, normalizing band intensities to the standard ladder (Novex™ Sharp Unstained Protein Standard LC5801, Thermo Fisher Scientific, USA).

### Quantitation of insoluble and soluble protein

For 1 mL protein expression, cells were lysed via a single freeze–thaw cycle in lysis buffer (50 mM sodium phosphate, 300 mM sodium chloride, 10 mM imidazole, 0.03% Triton X-100). The insoluble fraction (pelleted debris and proteins) was separated from the soluble fraction (supernatant) and resuspended in 8 M urea at equal volume to the soluble fraction. After 15 min incubation at room temperature, samples were centrifuged at 13,000 g for 30 min. Both fractions were mixed with Laemmli buffer (2 × Laemmli Sample Buffer #1610737EDU, Bio-Rad, USA), boiled at 95 °C for 5 min, and analyzed by SDS-PAGE (Thermo Fisher Scientific, USA). Gels were stained with InstantBlue^®^ Coomassie Protein Stain (Abcam, UK) for ≥ 60 min, destained for 60 min, and analyzed using Bio-Rad ImageLab software. Soluble protein was quantified as a percentage of total protein (soluble + insoluble) using densitometric data.

For protein expression at 10 mL and larger scales, cell pellets were resuspended in lysis buffer at a ratio of 1:5 (lysis buffer: culture volume) and lysed by sonication at 20% amplitude (10 s on/30 s off for 5 min 40 s, Qsonica). The lysate was clarified by centrifugation at 13,000*g*, and the insoluble fraction was processed and analysed as described for the small-scale expression.

### Statistical analysis

Data were analysed in Excel with one-way ANOVA. If there was statistical significance between all the sample groups (p < 0.05), a post-hoc Tukey HSD (Honestly Significant Difference) would be carried out to determine any statistical differences between every single pairing within the sample groups. The respective p-values for each pairing are displayed in the Figures. Error bars represent the sample standard deviation for biological triplicates.

## Results

### Alanine scanning of the 11 amino acid tag yields enhanced variants

**Fig. 1:**
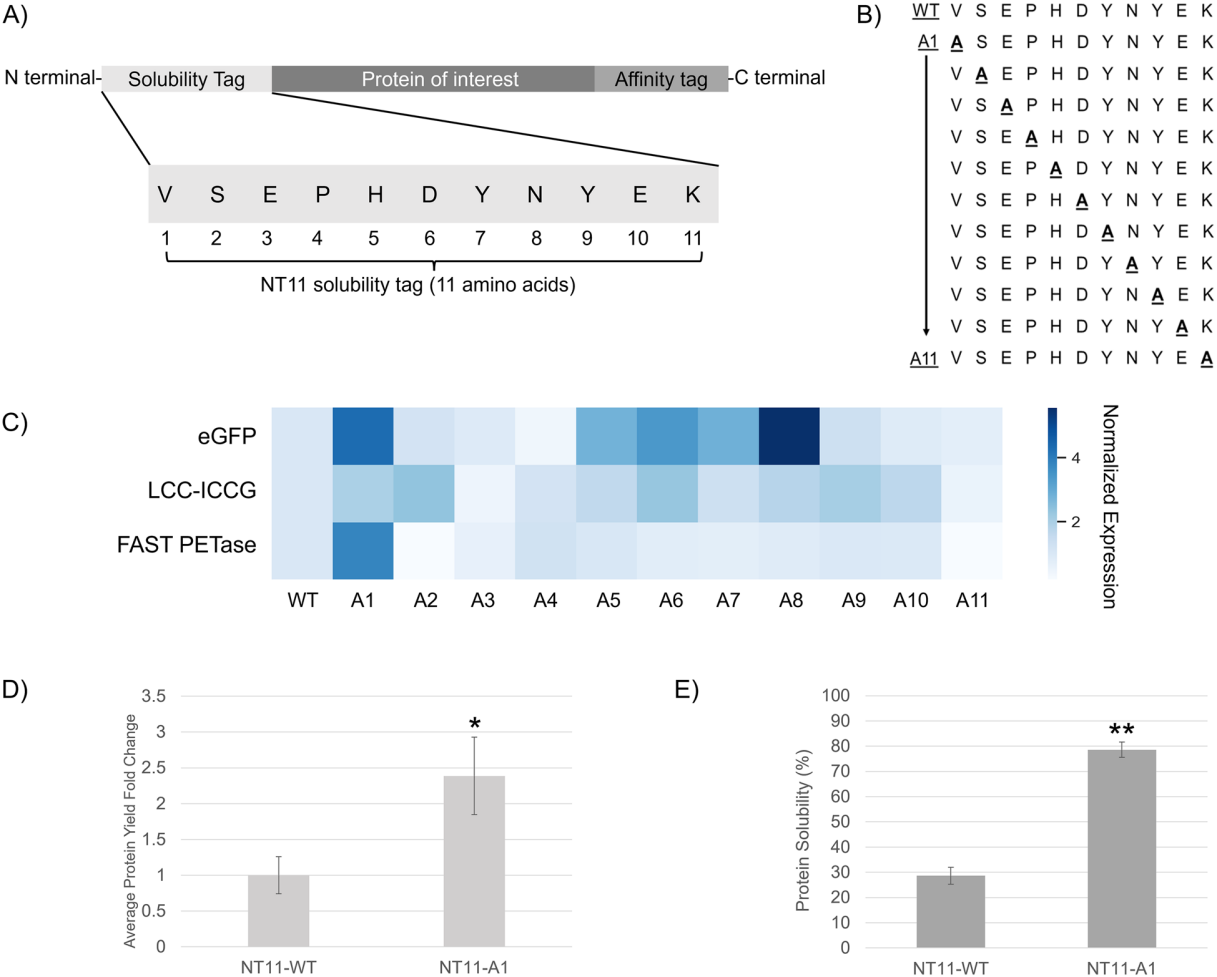
Alanine scan of the NT11 tag. **A** General protein schema for protein expression. **B** Alanine scan: A total of 11 NT11 mutants were generated via single alanine substitutions along the entire length of the protein tag. **C** Fold change in protein expression (densitometry) compared to wild type (WT) NT11 is represented as a heatmap featuring three proteins: the enhanced green fluorescence protein (eGFP [18]), the leaf-branch compost cutinase (LCC-ICCG [19]), and polyethylene terephthalate (PET [20]) plastic degrading enzyme (FAST PETase). The highest expression for each protein is highlighted in red squares with its corresponding fold-change displayed above in the same column. The fold-change in graphical format can be found in Figure S1. **D** Purified recombinant protein yield comparison between wildtype and A1 mutant of NT11-FAST PETase, with statistically significant (*p < 0.05) difference. Biological triplicates were performed (Figure S2). **E** Protein solubility is represented as a percentage comparing the wildtype tag to the NT11-A1 tag. The mutant tag resulted in statistically significant (**p < 0.01) differences in percentage solubility for FAST PETase. Biological triplicates were performed (Figure S3)

Given the short length of 11 amino acids, we hypothesise that substituting these amino acids may enhance the tag's effectiveness or help identify key residues for further engineering to create an improved tag. To examine this, we designed a general expression cassette with NT11 as a tag at the N-terminal (Fig. 1A). The NT11 tag and its mutants will be combined with fusion partners, and the resulting protein yield after expression will indicate the overall effectiveness of mutagenesis compared to the wildtype.

The single alanine substitution of each amino acid along the length of the NT11 tag produced 11 mutants, labelled NT11-A1 to A11 (Fig. 1B). A total of three proteins were paired with the NT11 tags and expressed to determine protein yield. The fluorescent protein eGFP (32.7 kDa, pI 6.2, [18]) is a common reporter protein for determining protein production while engineered versions of a Leaf Compost Cutinase (LCC-ICCG, 31.5 kDa, pI 9.3, [19]) and a PET degrading enzyme (FAST PETase, 27.6 kDa, pI 9.6, [20]) are both known plastic depolymerization enzymes involved in polyethylene terephthalate (PET) degradation and recycling. PET degrading enzymes are also known to have difficulty being expressed in *E. coli*, which is hypothesized to be due to their disulfide bridges [21–23].

From the screening of single alanine mutated tags, we observed that improved expression levels of up to fivefold can be achieved with a single mutation, however these were dependent on the specific protein pairing (Fig. 1C). Even so, we observed that A1 position consistently exhibit a two- to four-fold increase across the three proteins. The highest yields were achieved with different protein-tag pairings across eGFP, LCC-ICCG, FAST PETase (A8, A2 and A1 respectively-Fig. 1C). In addition to yield improvement, certain mutants also led to decreased protein yields, particularly at positions A3 and A11 (Fig. 1C), where no or reduced soluble yield was observed. These residues correspond to negatively charged glutamic acid and positively charged lysine respectively, suggesting the importance of having specific charges at their respective positions even in an 11-amino acid long peptide sequence.

To further clarify the impact of these mutations, additional solubility measurements were conducted, specifically for the NT11-A1 variant of FAST PETase, where we observed a 2.5-fold increase in purified recombinant protein yields (Fig. 1D, Figure S2). In further analyses of the insoluble and soluble fractions for NT11-FAST PETase and its corresponding improved mutant, we observed more than two-fold improvement in soluble/insoluble ratios with the mutated tag (Fig. 1E). Also of note, the proteins in the insoluble fractions run slower on the gel (Figure S3), which may be contributed to misfolding from its two disulfide bonds. In parallel, comparison of in silico prediction of free energy using the IUPred3 model (Figure S4) also predicted that the mutation of the first residue (A1) would create a disordered thus more soluble protein region at the N-terminal compared to that of the wildtype tag [24].

### NT11 tag as a flexible enhancement motif

**Fig. 2:**
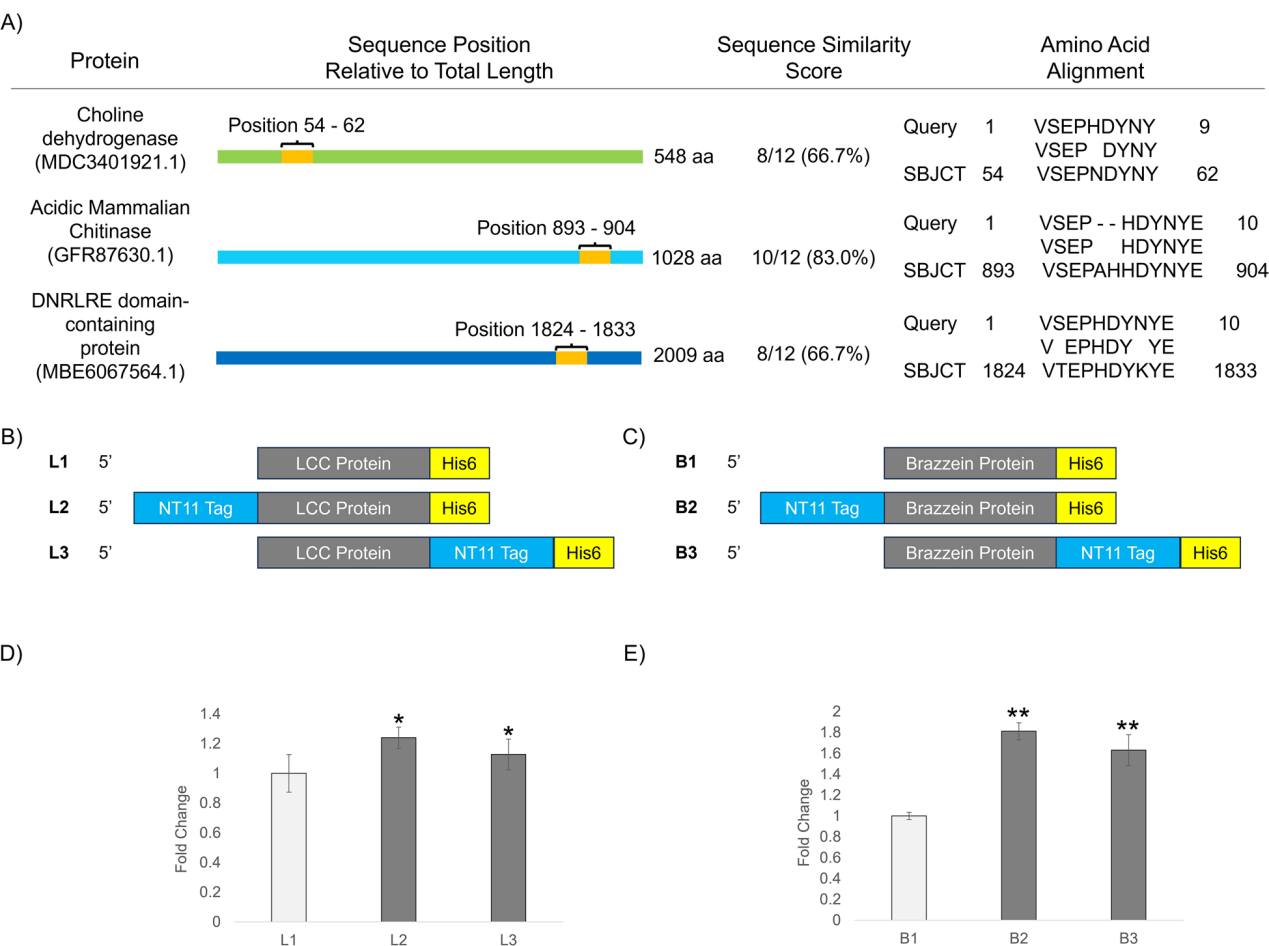
The flexibility of the NT11 tag and its ability to be placed on both ends of the protein while still functioning as an enhancement tag. **A** Properties of amino acid sequences, highly similar to NT11, that were found across various naturally occurring proteins. **B**, **C** Schematic representations of the three protein constructs designed for each target protein (LCC-ICCG and brazzein). **D**, **E** Fold change of soluble protein yields, with respect to the various NT11 placements, as calculated by densitometry. There were statistically significant (*p < 0.08, **p < 0.01) differences between the control and tagged samples but not between the tagged samples. Experiments were performed with biological triplicates

Upon performing in silico searches in Genbank using NT11 as a protein query, we discovered highly similar sequences (Fig. 2A) present in a variety of natural proteins. These proteins include enzymes such as mitochondrial transmembrane protein choline dehydrogenase and anti-pathogen acidic mammalian chitinase [25, 26], as well as structural proteins like DNRLRE-domain containing proteins [27, 28]. We discovered NT11-like sequences located not only near the N-terminal, as seen with dCA, but also close to the C-terminal in proteins such as acidic mammalian chitinase and DNRLRE-domain containing proteins. This discovery led us to hypothesize that the effect of the NT11 motif might not be confined to the prototypical N-terminus tag location.

To explore the potential placement of NT11 at different termini of a recombinant protein, we designed three sets of constructs using two proteins: the PET degrading enzyme LCC-ICCG (Fig. 2B, [19]) and the plant-derived sweet protein brazzein (Fig. 2C, [29]). These proteins contain two to four disulfide bonds and present exciting potential in applications of plastic recycling and alternative non-calorie sweeteners, where yields are essential to achieving cost efficiencies.

The expression constructs of the proteins proceeded with (1) no tag, (2) an NT11 tag placed at the N-terminal, and (3) an NT11 tag at the C-terminal. Additionally, all constructs have a C-terminal His-tag to facilitate affinity purification. In both LCC-ICCG and brazzein, we observed similar trends in the improvement of soluble recombinant protein yield with tags appended to either the N- or C-terminus of the target protein (Fig. 2D, E). To compare soluble protein yields, we compared purified protein yields. Here, we observed that the NT11 tag can improve yields when attached to either the N- or C-terminus of a paired protein, which is also bordered by affinity tags. To ensure that the addition of C-terminal tags does not affect protein folding, we also examined the specific PET depolymerisation activity of the various NT11-tagged LCC-ICCG at 70 °C. In LCC-ICCG, the thermal stability is contributed by its disulfide bond pairs; consequently, consistent activity at high temperatures of 70 °C (Table S3) suggests that disulfide bonds were correctly folded, in the presence of either N- or C- terminal tags.

### Improved expression of growth factors

**Fig. 3:**
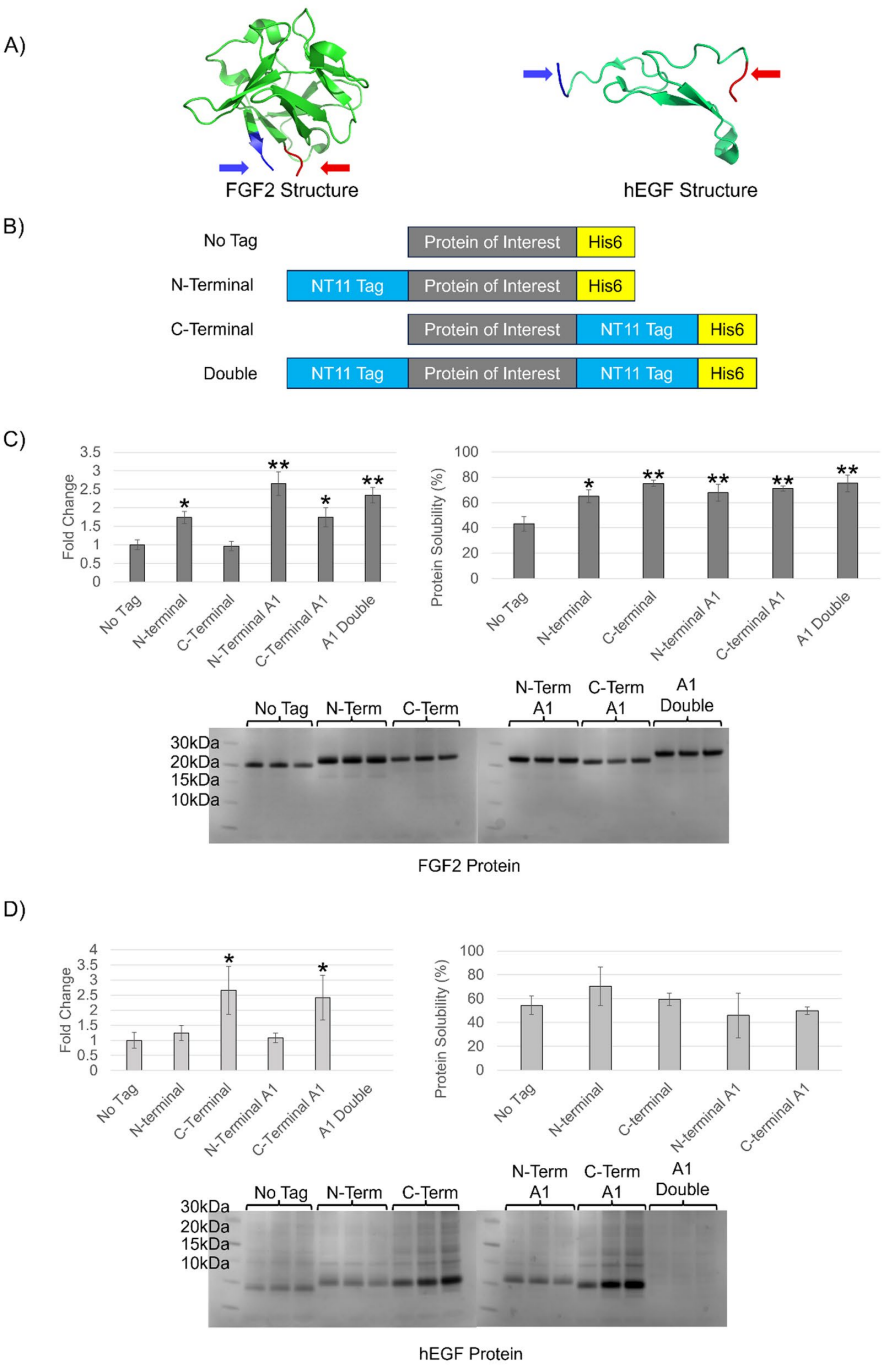
Growth factor expression under micro-bioreactor conditions. **A** Three-dimensional structure of FGF2 (PDB: 1BFB) [34] and hEGF (PDB: 1IXA) [35], with the N- and C-termini indicated in blue and red, respectively. **B** Schematic representation of the protein constructs used, with FGF2 and hEGF as the target proteins. **C**, **D** Comparison of fold change in soluble protein yields (left), protein solubility percentages (right), and corresponding SDS-PAGE gels of purified soluble proteins (below) for FGF2 (**C**) and hEGF (**D**). Fold-changes were calculated based on densitometric analysis of purified protein bands. All experiments were conducted in biological triplicates. Recombinant protein yield differed significantly (*p < 0.05, **p < 0.01) from the non-tagged control for all constructs, except the C-terminal tag for FGF2 and the N-terminal or N-terminal A1 tagged constructs for hEGF. Statistically significant differences (*p < 0.05, **p < 0.01) in solubility ratios relative to the non-tagged control were observed only for FGF2. For hEGF, one-way ANOVA revealed no statistically significant differences in solubility between groups. Triplicate solubility gel data can be found in Figures S5 and S6

Using a combination of NT11, NT11-A1 tags, and location placement, we investigated the effects of incorporating NT11 onto two structurally distinct growth factors, fibroblast growth factor 2 (FGF2) [30] and human epidermal growth factor (hEGF) [31]. While the two growth factors differ in sizes (~ 20 kDa and ~ 10 kDa for FGF2 and hEGF respectively) and their three-dimensional structures (Fig. 3A, Figure S2 [32, 33]), they are both small proteins (< 20 kDa),, making a small solubility tag like NT11 particularly suitable. For each growth factor, we investigated the effects of the NT11 tag using five constructs: (1) N-terminal tagged, (2) C-terminal tagged, (3,4) equivalent N- and C-terminal tagged for the NT11-A1 mutant and (5) NT11-A1 tagged at both the N and C-termini (Fig. 3B).

The purified growth factors showed distinct fold-change improvements over constructs without the NT11 tag. FGF2 protein demonstrated up to 2.6 ± 0.3-fold yield improvement over the non-tagged construct while hEGF demonstrated up to 2.6 ± 0.8-fold yield improvement (see Fig. 3). In further investigations into the effects of the tags on solubility, we observed two different scenarios. For FGF2, there is a general improvement in soluble/insoluble ratios with either C-terminal or N-terminal tagged NT11 or a doubly tagged NT11. For hEGF, although improvements in soluble recombinant protein yields are observed for C-terminal tagged variants, there were minimal changes in soluble/insoluble ratios. Under these conditions, the C-terminal tag has resulted in improvements in overall protein yields. It is worth noting that in this particular protein, combination of both C-terminal and N-terminal tags has resulted in a significant loss of soluble protein production.

## Discussions

Classical fusion tags (e.g. MBP, GST, NusA) have traditionally been essential for recombinant protein expression and are commonly used to address issues with insolubility [7]. However, these tags are typically large and often required removed in downstream processing, adding complexity. In recent years, the field has shifted toward smaller, more efficient fusion tags. These include short peptide sequences like the SKIK motif, which enhances translation efficiency [36], and intrinsically disordered fragments such as the NEXT tag which can improve solubility and yield [37]. NT11 belongs to this emerging category of minimalist tags that are part of an evolving paradigm in enhancing protein expression.

Our study demonstrated that specific single-point mutations in NT11 can significantly enhance protein expression—up to fivefold, depending on the target protein (Fig. 1). Among these, the A1 mutation stood out, consistently improving expression by two- to fourfold across multiple proteins. This highlights the importance of the N-terminal residue in the tag, which may influence translation efficiency and early folding. This observation is consistent with previous work showing that the identity of the first few amino acids, such as those in the SKIK peptide, can enhance ribosomal interactions and protein synthesis [36].

However, in our in silico calculations of NT11-A1, we also observed increased protein disorder, which led us to hypothesize an alternative explanation for NT11’s effectiveness. Similar to the mechanism proposed for the NEXT tag, NT11 may reduce aggregation through steric exclusion and entropic repulsion [37]. Notably, NT11 enhanced solubility when fused at either terminus of the target protein, suggesting a post-translational mechanism of action. Together, these observations support the idea that NT11 behaves predominantly as an intrinsically disordered tail, functioning without disrupting native protein folding.

Since NT11 retains its solubilizing effect when positioned at either the N- or C-terminus, it now offers a level of versatility, not commonly seen with traditional tags. Typically, solubility-enhancing tags are positioned at the N-terminus to function co-translationally. This N-terminus placement allows them to emerge early from the ribosome and recruit cellular chaperones during synthesis [38]. However, this approach can limit the flexibility of protein design, particularly for proteins where the N-terminus is crucial for proper function, folding, or interactions. In contrast, the adaptable placement of NT11 significantly enhances its versatility and applicability. Additionally, NT11's small size allows for seamless integration with affinity tags, such as His-tags, making it compatible with standard expression and purification workflows.

We also extended the application of NT11 towards high value bioproducts. FGF2 [30] and hEGF [31] are two high-value growth factors belonging to 2 separate growth factor families which garnered significant interest in recent years for their expression in prokaryotic systems in attempts to develop low costing, high yielding manufacturing routes [39, 40]. Due to their high demand in cell culture work and as treatment components for injuries, there were constant need for new avenues of production [39, 41]. A recently published work performed cost analysis of mitotic growth factors used in cell culture media formulations and found that currently, 1 mg of growth factor costs more than CAD$1000 (approx. USD$730 at time of writing) [41].

One effective cost reduction strategy is the utilization of microbial cell factories, such as *E. coli*. However, challenges like protein aggregation and low yields can arise. While solubility tags have been tested, their effectiveness varies and often involves complex purification processes. [40, 42]. FGF2 and hEGF contain two and three disulfide bonds respectively [43, 44]. When expressed in the bacterial cytoplasm, they often misfold into inclusion bodies or undergo rapid degradation. Conventional solutions, such as fusion with large chaperone tags (e.g., thioredoxin), protein refolding, or continuous biofilm-based production [39], are often labour-intensive and costly. Some of the most successful designs recorded demonstrated the expression of functional purified FGF2, achieving 100 mg/L yields upon the removal of tag [43]. On the other hand, recent efforts at expressing high-yielding hEGF made use of continuous free-cell cultures aided by biofilm formation to achieve yields up to 52.4 mg/L [39].

In contrast, we have shown NT11 to be a simple and effective alternative. The NT11 tag was sufficient to double the soluble recombinant protein expression yield, with FGF2 expression being improved further using mutant variant NT11-A1 to achieve at least 2.5-fold improvements. Compared to larger fusion tags like GST or MBP, NT11 offered equal or superior expression yields with significantly less metabolic burden. This is especially advantageous for small proteins such as hEGF, where bulky tags can disproportionately impact production efficiency and cost.

Our investigation into NT11 has expanded our knowledge and unveiled innovative strategies to boost recombinant growth. This study reveals that a straightforward alanine scan is a powerful and efficient preliminary screening tool for increasing protein yields; achieving improvements ranging from two to five times that of the wild-type tag. Additionally, it emphasises that the benefits of solubility tags are highly contingent upon the specific protein being studied. The differences observed in tag mutagenesis and positional effects, especially in growth factors with unique structures (Fig. 3), indicate that integrating various sites and employing in silico folding predictions, together with protein of interest, could significantly refine amino acid substitutions for optimal enhancement. Moreover, the remarkable versatility of NT11, which maintains its effectiveness regardless of its position when used in conjunction with an affinity tag such as a His-tag, paves the way for exciting new possibilities in the field of soluble recombinant protein production.

## Conclusions

Small solubility tags, such as NT11 tags, are highly attractive due to its size and impact. In this study, through alanine scanning and further characterisation of NT11, we expanded upon the utility of a short peptide tag. To demonstrate its utility, we tested NT11 on a diverse range of proteins with varied surface charges, structures and disulfide bonds (Figure S2) [45]. In the presence of an N-terminal or C-terminal tag or both, we observed improvements of over 60% in the proteins we examined. Due to the short length of the NT11 tag, alanine scanning was a simple and effective method to quickly enhance soluble recombinant protein yields.

Through further characterisation, we identified an additional property of NT11 based on our observations of similar NT11 sequences in nature. Here, we demonstrated the first examples of NT11 as an enhancer of soluble recombinant protein yields at both the N- and C-termini compared to the protein of interest. This flexibility opens up numerous possibilities for arranging protein-tag combinations, along with purification strategies.

With these new findings, we managed to double the recombinant production of soluble growth factors in *E. coli*. FGF2 expression could be further improved using NT11, with mutant variant NT11-A1 increasing expression by more than 2.5-fold. While novel methods have been employed for producing hEGF due to its low yield in *E. coli* [44], we have shown that simply utilizing the NT11 tag was sufficient to double the yield of soluble recombinant protein expression. Overall, the small size of NT11, coupled with its newly observed flexibility in placement and design, represents a valuable tag for use in efficient soluble recombinant protein expression and purification.

## Supplementary Information

Supplementary Material 1.

## Data Availability

No datasets were generated or analysed during the current study.

## Abbreviations

*E. coli*: *Escherichia coli*
FGF2: Fibroblast growth factor 2
hEGF: Human epidermal growth factor
SynIDPs: Small synthetic intrinsically disordered proteins
dCA: Duplicated carbonic anhydrase
LB: Luria Bertani
IPTG: Isopropyl-β-d-1-thiogalactopyranoside
IMAC: Immobilized metal affinity chromatography
eGFP: Enhanced green fluorescence protein
LCC: Leaf-branch compost cutinase
PET: Polyethylene terephthalate
