# Supplementary material for: Further characterization and engineering of an 11-amino acid motif for enhancing recombinant soluble protein expression
Source: Microb Cell Fact. 2025 May 24;24:122. doi: 10.1186/s12934-025-02738-5 (PMC12103771; doi:10.1186/s12934-025-02738-5)
Supplement: Supplementary file 1 — Supplementary Material 1. [file 12934_2025_2738_MOESM1_ESM.pdf]

Supplementary data

Further characterization and engineering of an 11-amino acid motif for enhancing recombinant soluble protein expression

Jiawu Bi[1,2], Elaine Tiong[1], Ying Sin Koo[3], Weibiao Zhou[2] and Fong Tian Wong [1, 3*]

[1] Institute of Molecular and Cell Biology (IMCB), Agency for Science, Technology and Research (A*STAR), 61 Biopolis Drive, Proteos #07-06, Singapore, 138673, Republic of Singapore
[2] Department of Food Science & Technology, National University of Singapore (NUS), Faculty of Science, 2 Science Drive 2, Singapore 117542, Republic of Singapore
[3] Institute of Sustainability for Chemicals, Energy and Environment (ISCE[2]), Agency for Science, Technology and Research (A*STAR), 8 Biomedical Grove, Neuros, #07-01, Singapore 138665, Republic of Singapore

*Corresponding Author: Fong Tian Wong
wongft@imcb.a-star.edu.sg

Figure S1. The protein expression fold change of each protein featured in the heatmap in Figure 1C presented in graphical format.

Figure S2. Protein gel for determining protein yield difference between FAST PETase NT11 WT and A1, used for calculating yield in Figure 1D in the main text.

Figure S3. Protein gel for alanine scan mutagenesis for determining protein solubility between wildtype and mutant constructs, A) used for calculating solubility percentage in Figure 1E in the main text.

Figure S4. Position-dependent prediction of protein disorder (IUPred3) comparing between two tagged FAST PETase proteins.

Figure S5. Protein solubility gels for FGF2 growth factor.

Figure S6. Protein solubility gels for hEGF growth factor.

Figure S7. The overall surface charge of eGFP (PDB: 6YLQ), LCC-ICCG (PDB: 4EB0), FAST PETase (7SH6), Brazzein (4HE7), FGF2 (PDB: 1BFB), hEGF (PDB: 1IXA) at pH 7.0 and the visualization of protein surface charge with negative charge (red) and positive charge (blue).

Table S1. Table documenting the full amino acid sequence of the proteins expressed with wild type NT11 tag.

Table S2. Table documenting the primer sequences used for performing QuikChange™ PCR mutagenesis for creating the alanine scan library of plasmids.

Table S3. Comparison of specific depolymerization activity of the variants in Figure 2D (% depolymerisation).

Supplementary data

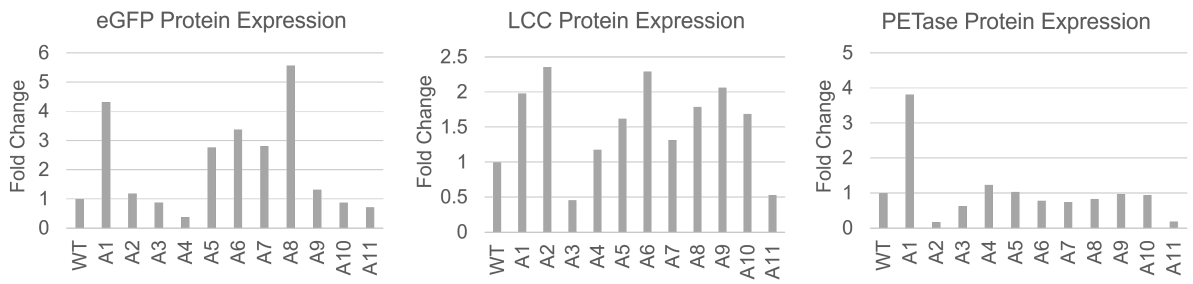

Figure S1. The protein expression fold change of each protein featured in the heatmap in Figure 1C presented in graphical format. High-throughput single pass experiment with no repeats was performed.

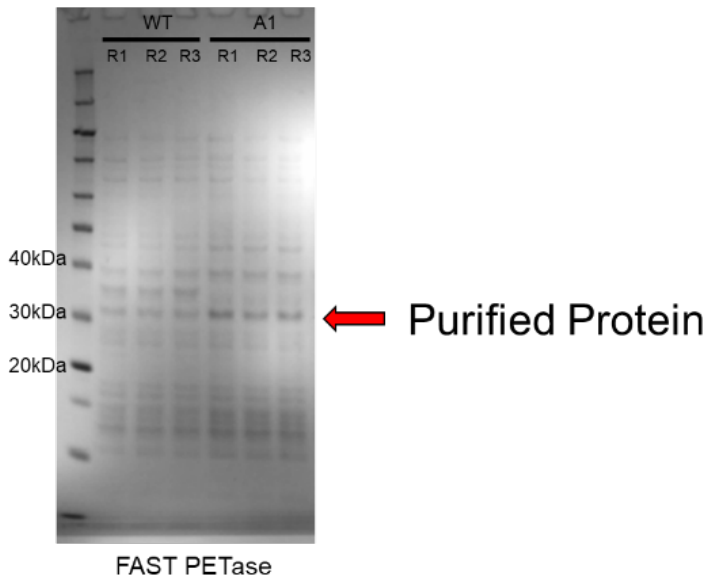

Figure S2. Protein gel of FAST PETase constructs featuring NT11 WT and NT11 A1 expressed at 1ml scale via IPTG induction and purified. It can be observed that even with His-tag purification, the wildtype construct retained some insoluble proteins above the 30kDa band. Samples were prepared in triplicates.

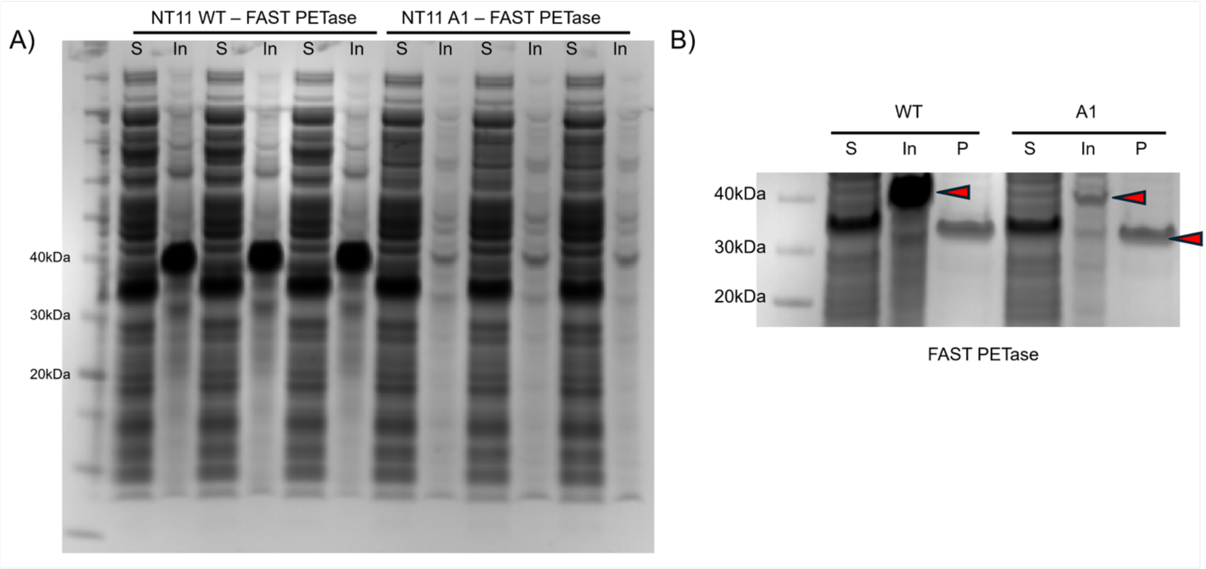

Figure S3. (A) Protein solubility gel of alanine scan mutagenesis for FAST PETase NT11 WT and NT11 A1 mutant. All protein samples for determining solubility were prepared in biological triplicates. B) A representative gel image showing the soluble (S), insoluble (In) and purified (P) fractions for FAST PETase with the WT NT11 tag and A1 NT11 mutant. Arrows indicate position of overexpressed FAST-PETase in the fractions.

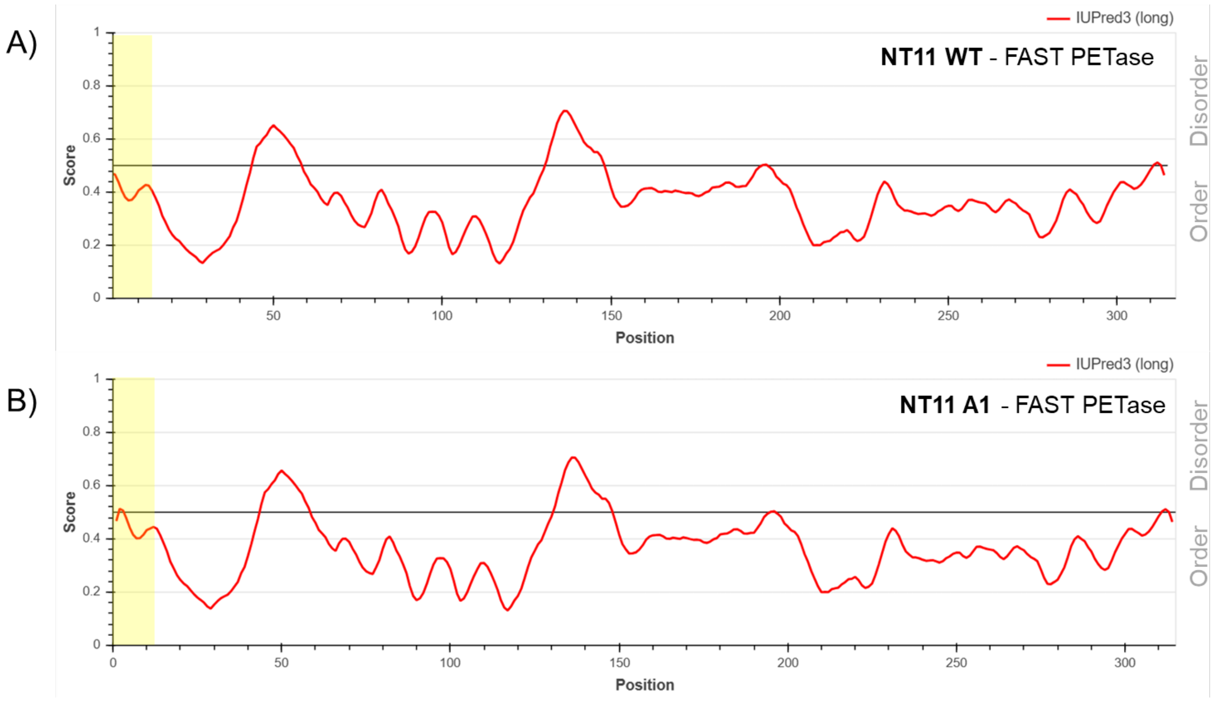

Figure S4. Position-dependent prediction of protein disorder (IUPred3) comparing between two tagged FAST PETase proteins. A) FAST PETase tagged with wildtype NT11 B) FAST PETase tagged with mutant A1 NT11. NT11 tag region highlighted in yellow.

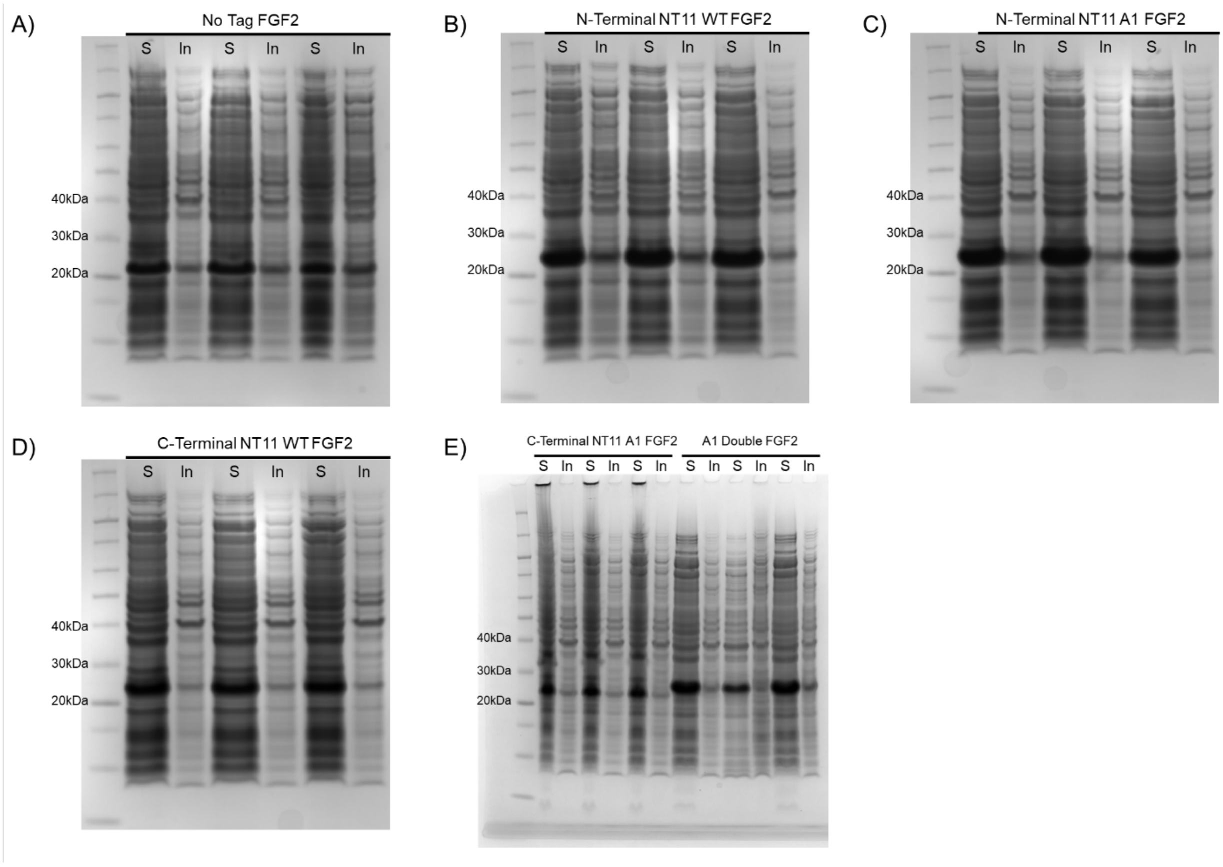

Figure S5. Protein solubility gels for FGF2 growth factor. A) No tag B) N-terminal WT tag C) N-terminal A1 tag D) C-terminal WT tag E) C-terminal A1 tag and A1 double tag. All samples prepared in biological triplicates

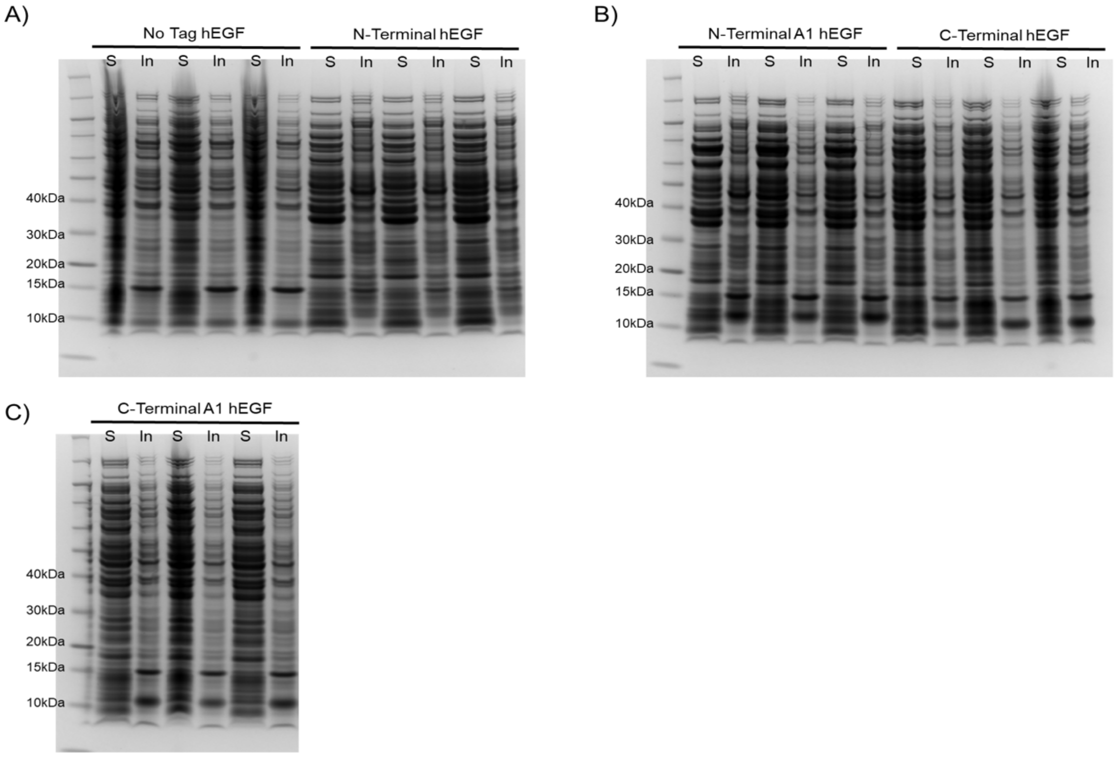

Figure S6. Protein solubility gels for hEGF growth factor. A) No tag and N-terminal WT tag B) N-terminal A1 and C-terminal WT tag C) C-terminal A1 tag. All samples prepared in biological triplicates

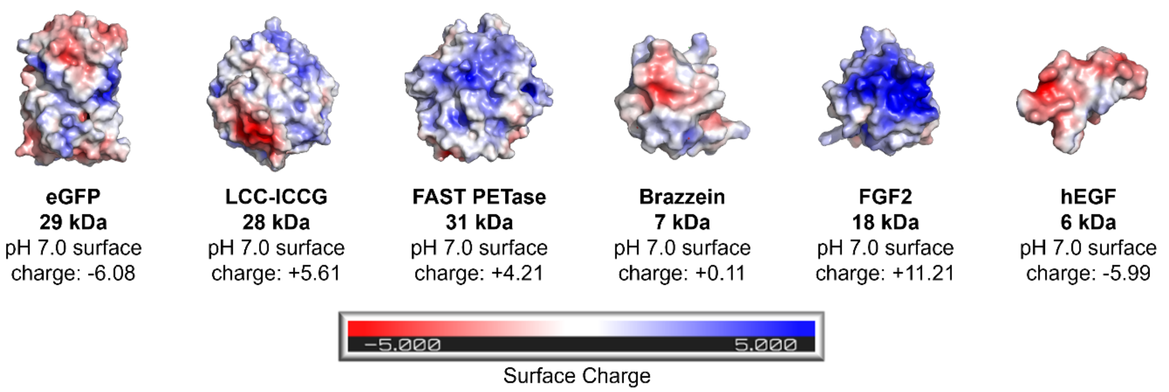

Figure S7. The overall surface charge of eGFP (PDB: 6YLQ), LCC-ICCG (PDB: 4EB0), FAST PETase (7SH6), Brazzein (4HE7), FGF2 (PDB: 1BFB), hEGF (PDB: 1IXA) at pH 7.0 and the visualization of protein surface charge with negative charge (red) and positive charge (blue).

Table S1. Table documenting the full amino acid sequence of the proteins expressed with wild type NT11 tag.

	Amino Acid Sequence	
Plasmid ID	Tag Sequence	Target protein sequence
pET28-NT11-eGFP	VSEPHDYNYEK	VSKGEELFTGVVPILVELDGDVNGHKFSVSGEGEGDATYGK LTLKFICTTGKLPVPWPTLVTTLTYGVQCFSRYPDHMKQHD FFKSAMPEGYVQERTIFFKDDGNYKTRAEVKFEGDTLVNRI ELKGIDFKEDGNILGHKLEYNYNSHNVYIMADKQKNGIKVNF KIRHNIEDGSVQLADHYQQNTPIGDGPVLLPDNHYLSTQSAL SKDPNEKRDHMVLLEFVTAAGITLGMDELYK
pET28-NT11-LCC	VSEPHDYNYEK	MSNPYQRGPNPTRSALTADGPFSVATYTVSRLSVSGFGGG VIYYPTGTSLTFGGIAMSPGYTADASSLAWLGRRLASHGFV VLVINTNSRFDGPDSRASQLSAALNYLRTSSPSAVRARLDA NRLAVAGHSMGGGGTLRIAEQNPSLKAAVPLTPWHTDKTF NTSVPVLIVGAEADTVAPVSQHAIPFYQNLPSTTPKVYVELC NASHIAPNSNNAAISVYTISWMKLWVDNDTRYRQFLCNVND PALCDFRTNNRHCQ
pET28-NT11-FAST_PETase	VSEPHDYNYEK	MNFPRASRLMQAAVLGGLMAVSAAATAQTNPYARGPNPTA ASLEASAGPFTVRSFTVSRPSGYGAGTVYYPTNAGGTVGAI AIVPGYTARQSSIKWWGPRLASHGFVVITIDTNSTLDQPESR SSQQMAALRQVASLNGTSSSPIYGKVDTARMGVMGHSMG GGGSLISAANNPSLKAAAPQAPWHSSTNFSSVTVPTLIFACE NDSIAPVNSSALPIYDSMSQNAKQFLEIKGGSHFCANSGNS NQALIGKKGVAWMKRFMDNDTRYSTFACENPNSTAVSDFR TANCS
pET28-NT11-Brazzein	VSEPHDYNYEK	MQDKCKKVYENYPVSKCQLANQCNYDCKLDKHARSGECF YDEKRNLQCICDYCEYP
pET28-NT11-FGF2	VSEPHDYNYEK	PALPEDGGSGAFPPGHFKDPKLLYCKNGGFFLRIHPDGRVD GTRDKSDPFIKLQLQAEERGVVSIKGVCANRYLAMKEDGRL YAIKNVTDECFFFERLEENNYNTYRSRKYPSWYVALKRTGQ YKLGSKTGPGQKAILFLPMSAKS
pET28-NT11-hEGF	VSEPHDYNYEK	NSDSECPLSHDGYCLHDGVCMYIEALDKYACNCVVGYIGER CQYRDLKWWELR

Table S2. Table documenting the primer sequences used for performing QuikChange™ PCR mutagenesis for creating the alanine scan library of plasmids.

Plasmid ID	Primer Nucleotide Sequence (5' to 3')
pET28-p538-NT11-A1	Forward: TGCGGTTCGGACGCcatGGTATATCTCCTT<br>Reverse: AAGGAGATATACCatgGCGTCCGAACCGCA
pET28-p538-NT11-A2	Forward: ATAATCATGCGGTTCCGCTACcatGGTATATCTCC<br>Reverse: GGAGATATACCatgGTAGCGGAACCGCATGATTAT
pET28-p538-NT11-A3	Forward: AGTTATAATCATGCGGCGCGGATACcatGGT<br>Reverse: ACCatgGTATCCGCGCCGCATGATTATAACT
pET28-p538-NT11-A4	Forward: CATAGTTATAATCATGCGCTTCGGATACcatGG<br>Reverse: CCatgGTATCCGAAGCGCATGATTATAACTATG
pET28-p538-NT11-A5	Forward: TGCCTTCTCATAGTTATAATCcgcCGGTTCGGATACca<br>Reverse: tgGTATCCGAACCGGCGGATTATAACTATGAGAAGGCA
pET28-p538-NT11-A6	Forward: TGCCTTCTCATAGTTATAcgcATGCGGTTCG<br>Reverse: CGAACCGCATgcgTATAACTATGAGAAGGCA
pET28-p538-NT11-A7	Forward: CTGCCTTCTCATAGTTcgcATCATGCGGTTCG<br>Reverse: CGAACCGCATGATgcgAACTATGAGAAGGCAG
pET28-p538-NT11-A8	Forward: CCTGCCTTCTCATAcgcATAATCATGCGGTTC<br>Reverse: GAACCGCATGATTATgcgTATGAGAAGGCAGG
pET28-p538-NT11-A9	Forward: CTCCTGCCTTCTCcgcGTTATAATCATGCGGT<br>Reverse: ACCGCATGATTATAACgcgGAGAAGGCAGGAG
pET28-p538-NT11-A10	Forward: GCTCCTGCCTTcgcATAGTTATAATCATGCG<br>Reverse: CGCATGATTATAACTATgcgAAGGCAGGAGC
pET28-p538-NT11-A11	Forward: cAGCTCCTGCcgcCTCATAGTTATAATCATGC<br>Reverse: GCATGATTATAACTATGAGgcgGCAGGAGCTg

Table S3. Comparison of specific depolymerization activity of the variants in Figure 2D (% depolymerisation). Reaction condition consist of incubation of 2 mg lcPET (<150 µm) and 1.67 µM purified proteins, in 100 mM potassium phosphate, pH 8, 70°C, 500 rpm and 3 hours. The experiment was conducted in triplicates.

LCC Activity Assay	**Enzyme**		
Depolymerization Products	**L1**	**L2**	**L3**
TPA	25.6 ± 1.93	29.1 ± 1.02	26.6 ± 2.03
MHET	34.4 ± 2.07	34.7 ± 0.29	32.6 ± 3.81
BHET	1.2 ± 0.06	1.1 ± 0.02	1.1 ± 0.13
Total depolymerized	61.2	65.0	60.0
